# *Gundelia tournefortii*: Fractionation, Chemical Composition and GLUT4 Translocation Enhancement in Muscle Cell Line

**DOI:** 10.3390/molecules26133785

**Published:** 2021-06-22

**Authors:** Sleman Kadan, Sarit Melamed, Shoshana Benvalid, Zipora Tietel, Yoel Sasson, Hilal Zaid

**Affiliations:** 1 Qasemi Research Center, Al-Qasemi Academic College, Baqa El-Gharbia 30100, Israel; slemanka@gmail.com; 2 Casali Center for Applied Chemistry, Institute of Chemistry, The Hebrew University of Jerusalem, Givat Ram, Jerusalem 91904, Israel; ysasson@huji.ac.il; 3 Department of Food Science, Gilat Research Center, Agricultural Research Organization—Volcani Institute, M.P. Negev 8531100, Israel; saritm@volcani.agri.gov.il (S.M.); tietel@volcani.agri.gov.il (Z.T.); 4 Regional Research and Development Center, Judea Center, Kiryat Arba 90100, Israel; shoshana.benvalid@mail.huji.ac.il; 5 Faculty of Sciences and Faculty of Medicine, Arab American University, P.O. Box 240, Jenin 009704, Palestine

**Keywords:** *Gundelia tournefortii*, GLUT4, fractionation, diabetes mellitus, phytochemicals

## Abstract

Type 2 diabetes (T2D) is a chronic metabolic disease, which could affect the daily life of patients and increase their risk of developing other diseases. Synthetic anti-diabetic drugs usually show severe side effects. In the last few decades, plant-derived drugs have been intensively studied, particularly because of a rapid development of the instruments used in analytical chemistry. We tested the efficacy of *Gundelia tournefortii* L. (GT) in increasing the translocation of glucose transporter-4 (GLUT4) to the myocyte plasma membrane (PM), as a main strategy to manage T2D. In this study, GT methanol extract was sub-fractionated into 10 samples using flash chromatography. The toxicity of the fractions on L6 muscle cells, stably expressing GLUTmyc, was evaluated using the MTT assay. The efficacy with which GLUT4 was attached to the L6 PM was evaluated at non-toxic concentrations. Fraction 6 was the most effective, as it stimulated GLUT4 translocation in the absence and presence of insulin, 3.5 and 5.2 times (at 250 μg/mL), respectively. Fraction 1 and 3 showed no significant effects on GLUT4 translocation, while other fractions increased GLUT4 translocation up to 2.0 times. Gas chromatography–mass spectrometry of silylated fractions revealed 98 distinct compounds. Among those compounds, 25 were considered anti-diabetic and glucose disposal agents. These findings suggest that GT methanol sub-fractions exert an anti-diabetic effect by modulating GLUT4 translocation in L6 muscle cells, and indicate the potential of GT extracts as novel therapeutic agents for T2D.

## 1. Introduction

Type 2 diabetes (T2D) is one of the leading causes of early mortality and morbidity globally, and is associated with various critical complications including, but not limited to, neuropathy, cardiovascular diseases [1] and hyperuricemia [2,3]. According to the World Health Organization (WHO), more than 422 million people worldwide were diabetic in 2014, and this number is expected to double in 2040. The prevalence of diabetes is the highest in the Middle East (13.7% in 2014), where the number of diabetic patients reached 43 million in 2014 [4,5]. T2D is mainly characterized by insulin resistance in hepatocytes, adipocytes, and myocytes [6,7]. Insulin resistance in skeletal muscle plays a critical role in the development of T2D. Insulin-stimulated glucose uptake into muscle cells accounts for approximately 75% of whole-body insulin-activated glucose disposal and nutrient utilization. In healthy subjects, insulin stimulates the mobilization of glucose transporter type 4 (GLUT4) to the surface of muscle fibers, and thereby enhances glucose uptake. Unfortunately, this process is impaired in patients with insulin resistance and diabetes [6].

The GLUT4 protein is expressed in cardiac and skeletal muscles, as well as brown and white adipocytes [6]. Unlike most other cells in which GLUTs are unregulated, GLUT4 distribution on the surface of muscle, liver, and fat cells is rapidly up-regulated several times in response to insulin and exercise. This process is known as GLUT4 translocation, as it largely requires changes in the distribution of GLUT4 between intracellular compartments and the plasma membrane (PM) [6,7].

Both T2D and insulin resistance can be prevented and managed by maintaining a healthy lifestyle. In addition, natural medicine, one of the therapeutic approaches of complementary and alternative medicine, has attracted considerable attention from individuals with T2D, especially because of its low cost and minimal side effects. Many of the active phytochemicals in herbs, especially polyphenolic compounds, are reportedly anti-diabetic agents [4].

*Gundelia tournefortii* L. (GT), a vegetable that is similar to artichoke, grows in the semi-arid climate of many countries in the Mediterranean region. Its common name is tumble thistle, also known as Tournefort’s gundelia, and it belongs to the Asteraceae or Compositae family. It is a spiny perennial, which grows to 30 cm in height, and may reach as high as 50 cm. The parts above the surface of the soil may break from the root and be blown away by the wind as tumbleweed, thereby facilitating seed dispersal. As a wild edible plant, GT has therapeutic effects against bacteria, cancer, epilepsy, stomach disorders, and diabetes [8,9].

The anti-diabetic activity of GT was evaluated in vivo in dexamethasone-induced diabetic mice. Oral administration of GT in diabetic mice led to significantly reduced levels of serum glucose [10]. Concomitantly, GT extracts displayed anti-diabetic activity in vitro as they enhanced GLUT4 translocation to the PM [9].

Using gas chromatography–mass spectrometry (GC/MS) analysis, we previously reported 44 new compounds among GT methanol and hexane extracts [9]. Sterols, esters, phenolic compounds, saturated and unsaturated fatty acids, and aromatic compounds were all detected. Only six components, namely, stigmasterol, β-sitosterol, linoleic acid, α-linolenic acid, stearic acid, and palmitic acid, have been previously reported [9,11]. Herein, we evaluated the efficacy of 10 sub-fractions of methanol GT extract in promoting GLUT4 entry into the PM of muscle cells. The chemical composition of the 10 fractions was identified, and their potential anti-diabetic mechanisms of action are herein reported.

## 2. Materials and Methods

Materials: The rat L6 muscle cell line, stably expressing myc-tagged GLUT4 (L6-GLUT4myc cells), was obtained from Kerafast (Boston, MA, USA). Fetal bovine serum, modified Eagle’s medium (α-MEM), standard culture medium, and all other tissue culture reagents were purchased from Biological Industries (Beit Haemek, Israel). Horseradish peroxidase (HRP)-conjugated goat anti-rabbit antibodies were obtained from Promega (Madison, WI, USA). Polyclonal anti-myc (A-14); the 3-(4,5-dimethylthiazol-2-yl)-2,5-diphenyl tetrazolium bromide (MTT) reagent; methoxyamine hydrochloride; pyridine; N-methyl-*N*-(trimethylsilyl)-trifluoroacetamide (MSTFA); and other standard chemicals were purchased from Sigma Aldrich (St. Louis, MO, USA).

### 2.1. Plant Extract Preparation

The aerial parts of the GT plant were collected from the northern Negev area in Israel from early March 2018 (the geographic coordinates (latitude and longitude) are 30°30′0.00″ N, 34°55′1.20″ E). The air-dried aerial parts (200 g) of the GT plant were powdered and mixed with 1 L of methanol and placed in an Erlenmeyer flask. The mixture in the Erlenmeyer flask was sonicated for 2 h at 50 °C, and then left in dark glass bottles for 24 h for complete extraction, to yield a dark green extract. The extract was filtered and concentrated by a rotary vacuum evaporator. The yield of the extracts was 6.1%. The stock extracts were maintained at −20 °C in an airtight glass container.

### 2.2. Flash Chromatography Extract Fractionation

The GT crude extract was further purified by normal phase column chromatography in a Teledyne ISCO flash chromatography system (Lincoln, NE, USA), equipped with an ultraviolet detector. The solvent was evaporated under reduced pressure, and the residue was dissolved in ethanol. About 3 g of silica, 40–60 µm, was added to the mixture. The solvent was evaporated under reduced pressure, and the residue was transferred to a cartridge and connected to a gold silica 40 g column (Teledyne ISCO). The chromatographic separation was performed in a RediSep Column (Teledyne ISCO). The mobile phase gradient comprised hexane: ethyl acetate: ethanol at a 40 mL/min flow rate, as shown in Table 1. Wavelengths of 254 nm and 210 nm were used for detection. Ten samples were collected.

### 2.3. Silylation Derivatization

A volume of 40 µL of 40 mg/mL methoxyamine hydrochloride (MeOX) solution in pyridine was added to each dried sample (10 mg), with 10 µL of ribitol standard solution (0.2 mg/mL). The samples were then shaken for 1.5 h at 37 °C, after which 90 µL of MSTFA was immediately added to each sample cap and shaken at maximum speed for 30 min at 37 °C. The contents were transferred to glass vials with micro-serts and immediately capped [12]. Each derivatized sample (1 µL) was injected into the gas chromatograph coupled with a mass spectrometer.

### 2.4. Gas Chromatography–Mass Spectrometry Analysis

GC/MS analysis was performed on a Agilent 6850 GC, equipped with Agilent 5975C single quadrupole MS, CTC-PAL RSI 85 auto-sampler, and HP-5MS capillary column (0.25 µm × 30 m × 0.25 mm). The following conditions were applied: injector temperature, 250 °C; initial temperature, 50 °C for 5 min; gradient of 5 °C/min until 180 °C; gradient of 10 °C/min until 270 °C and a hold time of 10 mi., and increasing to 320 °C. The MS parameters were set as follows: source temperature, 230 °C; transfer line, 325 °C; quadrupole: 150 °C; Detector: 325; positive ion monitoring; and electron ionization (EI)-MS measurement at 70 eV [12]. Helium was used as a carrier gas, at 0.6 mL/min.

### 2.5. Identification of Components

The percentage composition of the samples was computed from the GC peak areas. Library searches were conducted using the National Institute of Standards and Technology GC/MS Library and mass spectra from the literature. Component relative percentages were calculated based on GC peak areas without using correction factors [12]. The GC/MS chromatogram is presented in Supplementary Figure S1.

Cell culture: The L6-GLUT4myc cells were grown in a humidified atmosphere of air and 5% CO_2_ at 37 °C. Myoblasts were maintained in α-MEM supplemented with 10% fetal bovine serum (FBS), 100 U/mL penicillin, and 0.1 mg/mL streptomycin.

### 2.6. MTT Assay

The L6-GLUT4myc cells were seeded in 96-well plates at a density of 20,000 cells per well, in the presence of increasing concentrations of GT fractions (0–500 µg/mL) for 24 h. The viability of those cells was evaluated by performing the MTT assay [13]. The culture medium was replaced with 200 µL of 0.5 mg/mL fresh MTT medium in each well, and cells were cultivated for another 4 h in an incubator within a dark room. The MTT medium was removed and 100 µL isopropanol was added to each well. The color density was measured at 620 nm with a microplate reader (Anthos, Biochrom, Cambridge, UK). The effects of the extracted fraction on cell viability were expressed using the following formula:

Percent viability = (A620 nm of fraction treated sample/A620 nm of untreated sample) × 100

### 2.7. Determination of Surface GLUT4myc

The GLUT4myc distribution on the PM of intact cells was assayed as previously described [13,14]. Briefly, cells were grown in 24-well plates for 1 day, after which GT extract fractions were added and left for 20 h. The mixtures were serum-starved for 3 h (in the presence of GT extract) and then treated either with or without 100 nM insulin for 20 min. The cells were washed with ice-cold phosphate-buffered saline (PBS), and then fixed with paraformaldehyde, and blocked with goat serum. The anti-myc antibody was added to the mixture, which was left for 1 h at 4 °C. The cells were again washed, and the secondary antibody conjugated to horseradish peroxidase was added and left for 1 h at 4 °C. The cells were then washed with PBS. We were able to detect and compare the relative amounts of GLUT4 on the PM of treated cells with those on the PM of vehicle-untreated cells. The *o*-phenylenediamine dihydrochloride reagent was used to develop the color, which was measured at 492 nm.

### Statistical Analysis

Results were presented as the mean ± SEM. Error limits were cited, and error bars were plotted and represent simple standard deviations of the mean. When comparing different samples, results were considered to be statistically different when *p* < 0.05. Data were analyzed using *t*-test statistical calculations conducted with the SPSS version 21.0 software.

## 3. Results

Using GC/MS analysis, we previously reported 44 new compounds among methanol and hexane extracts of the aerial parts of GT. Sterols, esters, phenolic compounds, saturated and unsaturated fatty acids, and aromatic compounds were detected [9]. To determine the anti-diabetic effects of GT methanol extracts and the potential anti-diabetic active fractions/phytochemicals, 10 sub-fractions were prepared from GT methanol extracts. The anti-diabetic effects of the sub-fractions were evaluated in L6-GLUT4myc cells.

### 3.1. GT Methanol Extract Fractionation and Chemical Detection

GT methanol extract was sub-fractioned into 10 samples via flash chromatography, as described in the methods section. To maximize the number of detected compounds, the fractions were silylated before GC/MS analysis.

**Figure 1:**
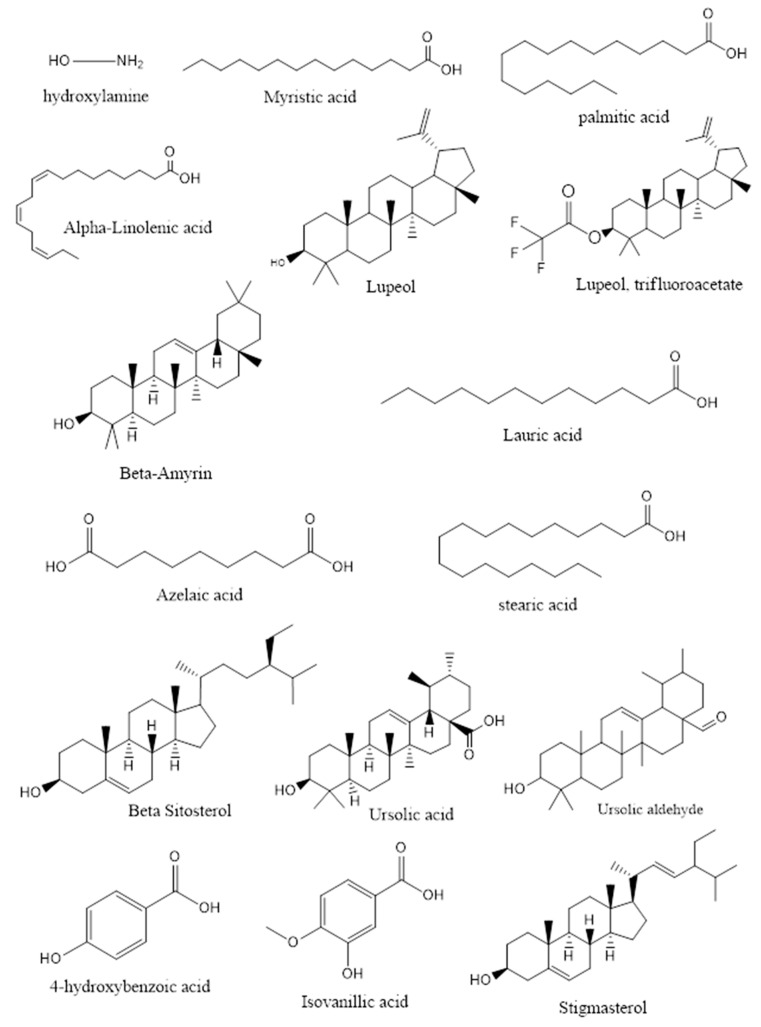

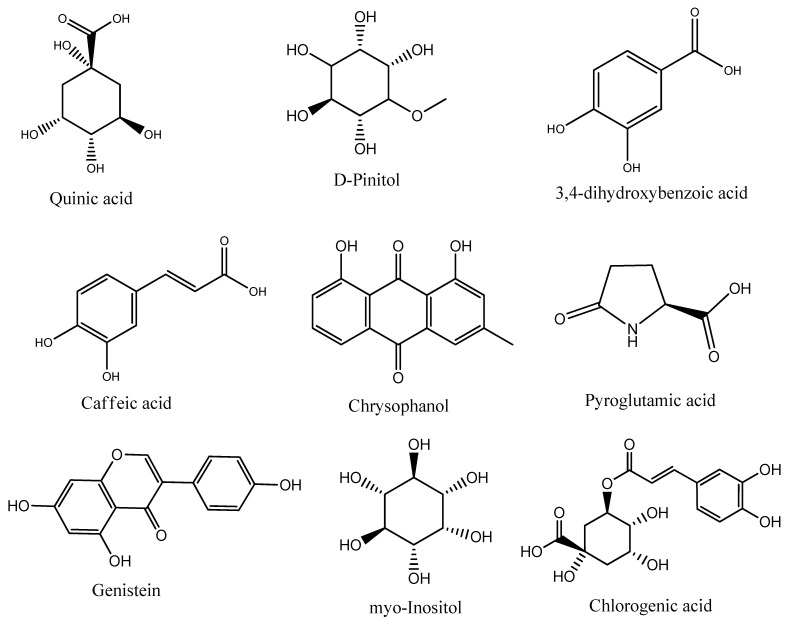
Chemical structure of the anti-diabetic and GLUT4 translocation enhancer phytochemicals exciting in the 10 GT fractions.

The compounds detected in fractions 1 to 10 (ranging from 3 to 29 compounds) are listed in Table 2, and their potential anti-diabetic activity is presented. The main compounds in each fraction are highlighted in bold (Table 2) and mentioned in Supplementary Figure S1. The structures of the phytochemicals known to exhibit anti-diabetic activity and enhance GLUT4 activity are shown in Figure 1.

### 3.2. Toxicity of GT Fractions and Effects on GLUT4 Translocation

**Figure 2:**
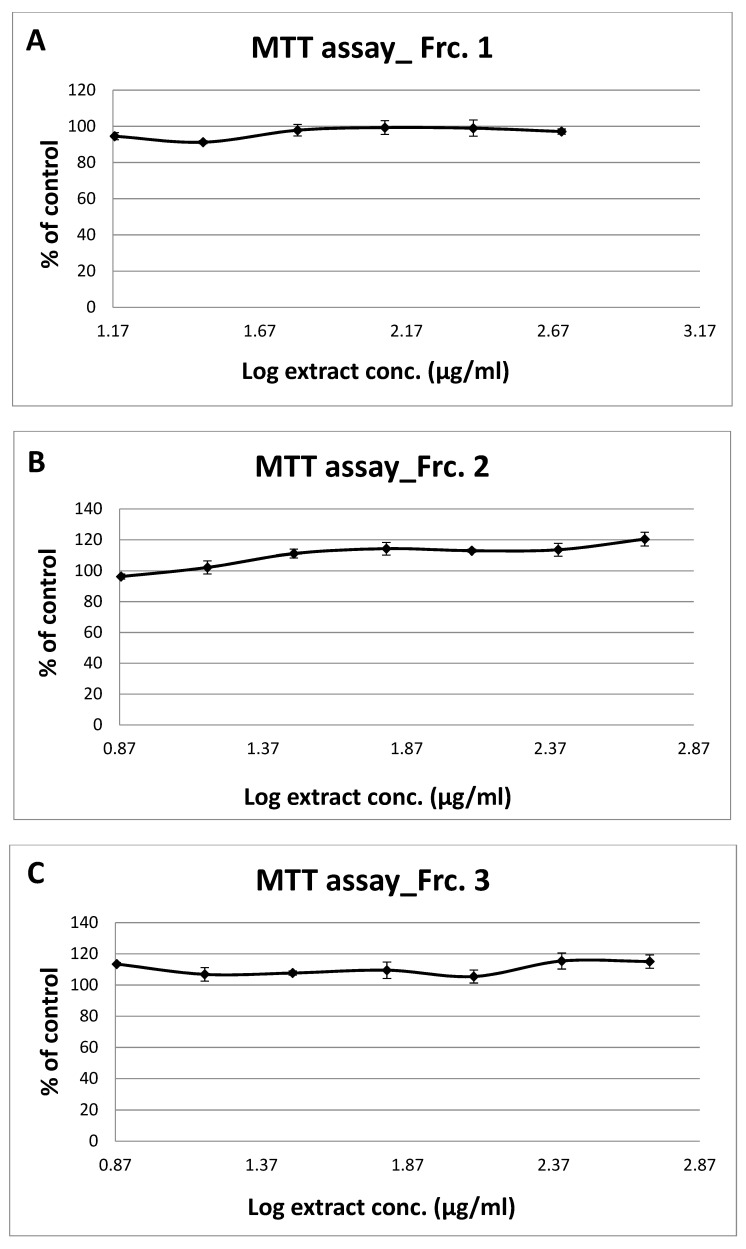

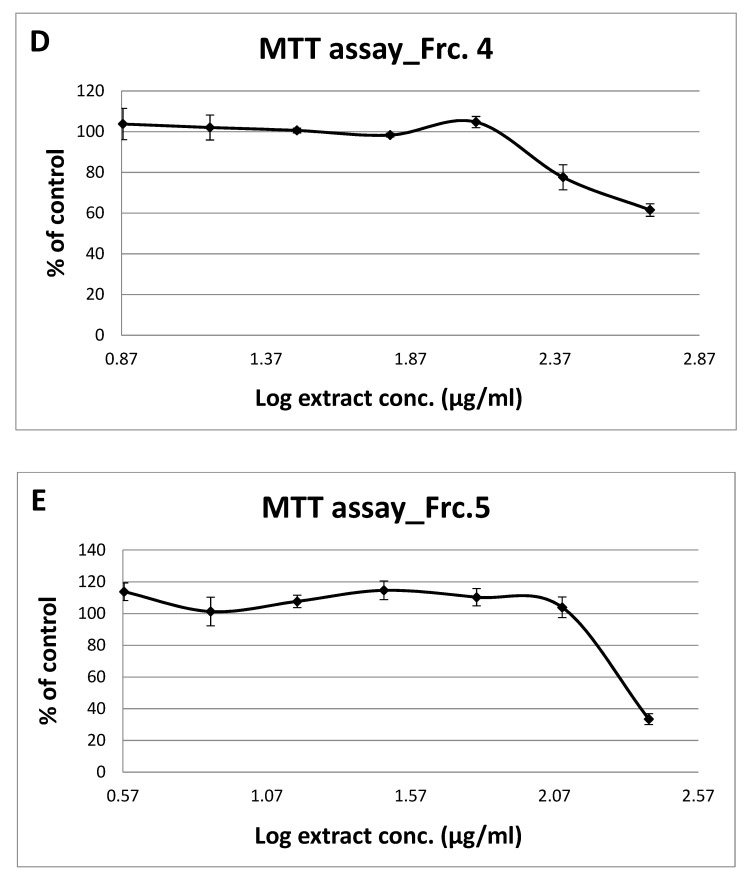

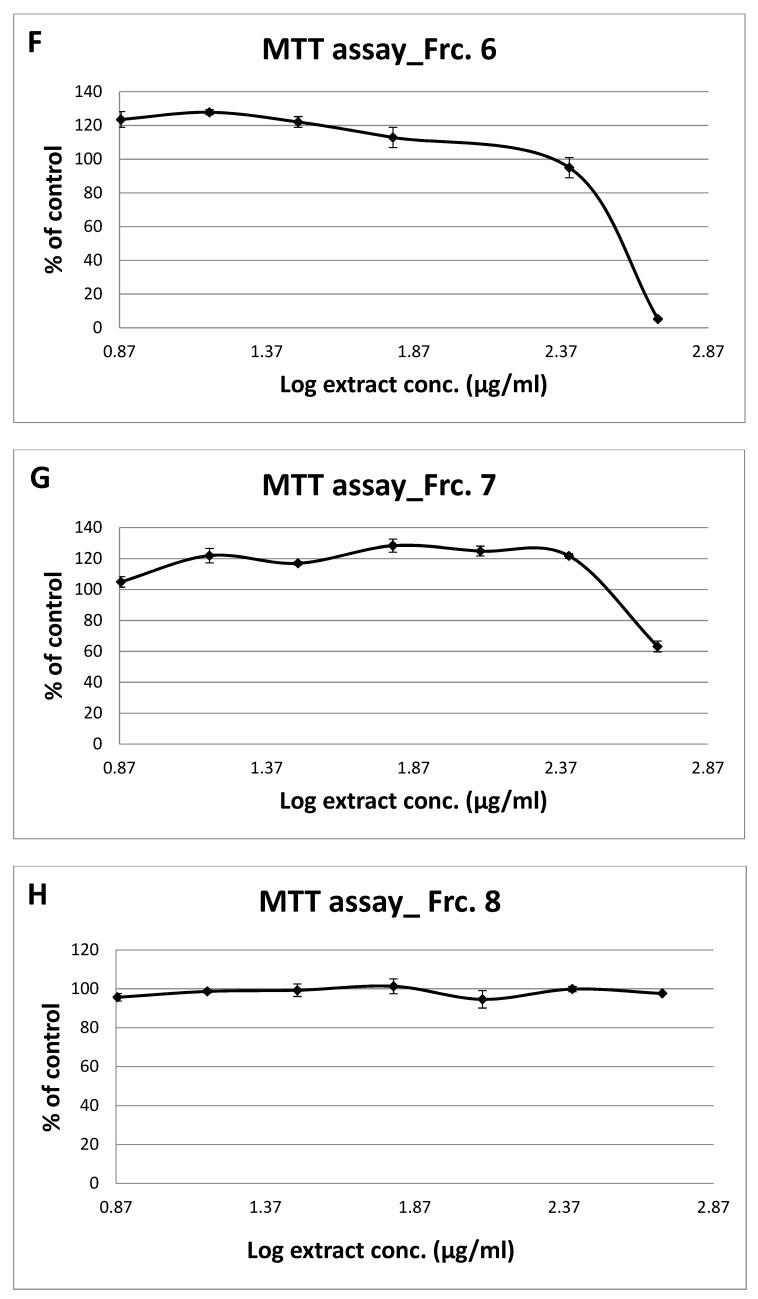

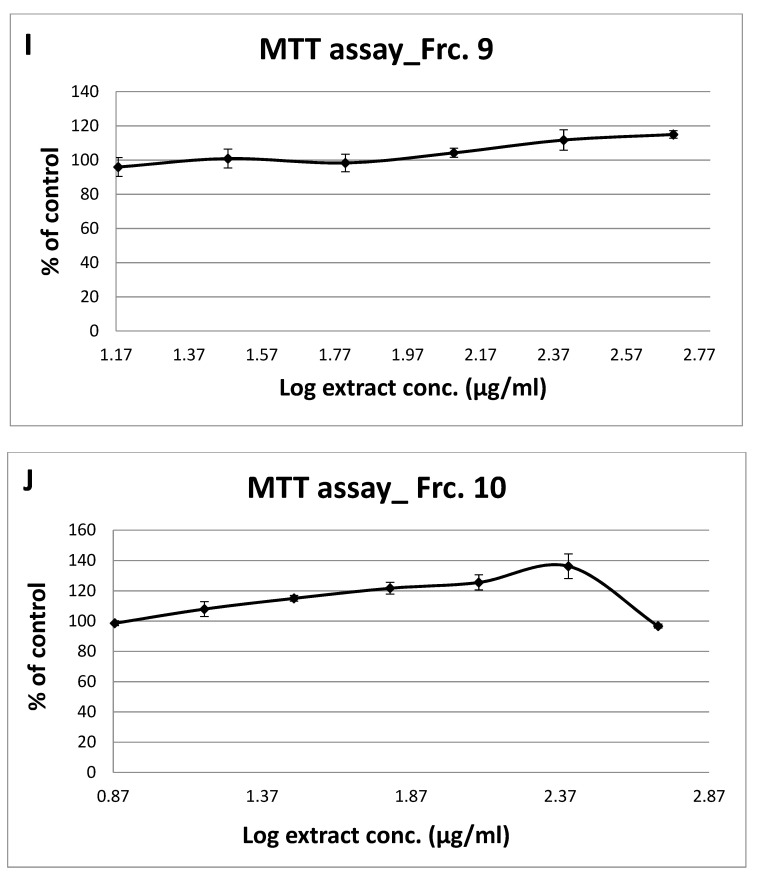
Effect of the Ten *Gundelia T.* fractions (**A**–**J**) on cell viability by MTT assay. L6-GLUT4myc cells (20,000 cell/well) and exposed to GT fractions for 20 h. Values given represent means ± SEM (% of untreated control cells) of three independent experiments carried out in triplicates.

The MTT assay was adopted to assess the toxicity of fractions on the L6-GLUT4myc cells. The cells were exposed to increasing concentrations of the GT extract fractions up to 500 µg/mL for 24 h. No toxic effects were observed for: fractions 1 to 3, and 8 to 10, up to 500 µg/mL; as well as fraction 5, up to 125 µg/mL; and fractions 4, 6, and 7, up to 250 µg/mL (Figure 2 and Table 3). To assess the efficacy of the fractions in promoting GLUT4 translocation to the muscle cell membrane, the L6-GLUT4myc cells were incubated with 125 and 250 μg/mL of each fraction for 23 h, both in the presence and absence of insulin. (Fractions 9 and 10 were evaluated at concentrations of 63 and 125 μg/mL, respectively, owing to the limitation of their fractionated volumes).

**Figure 3:**
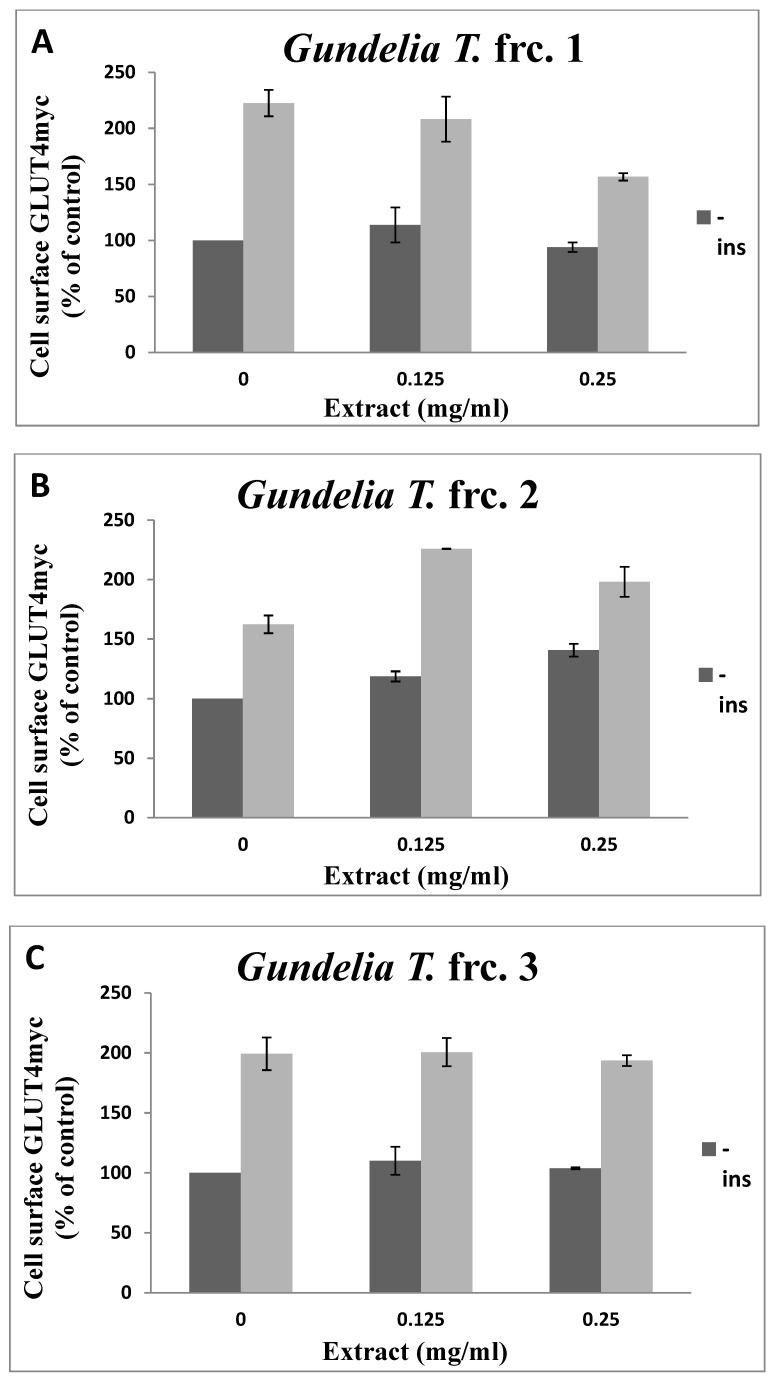

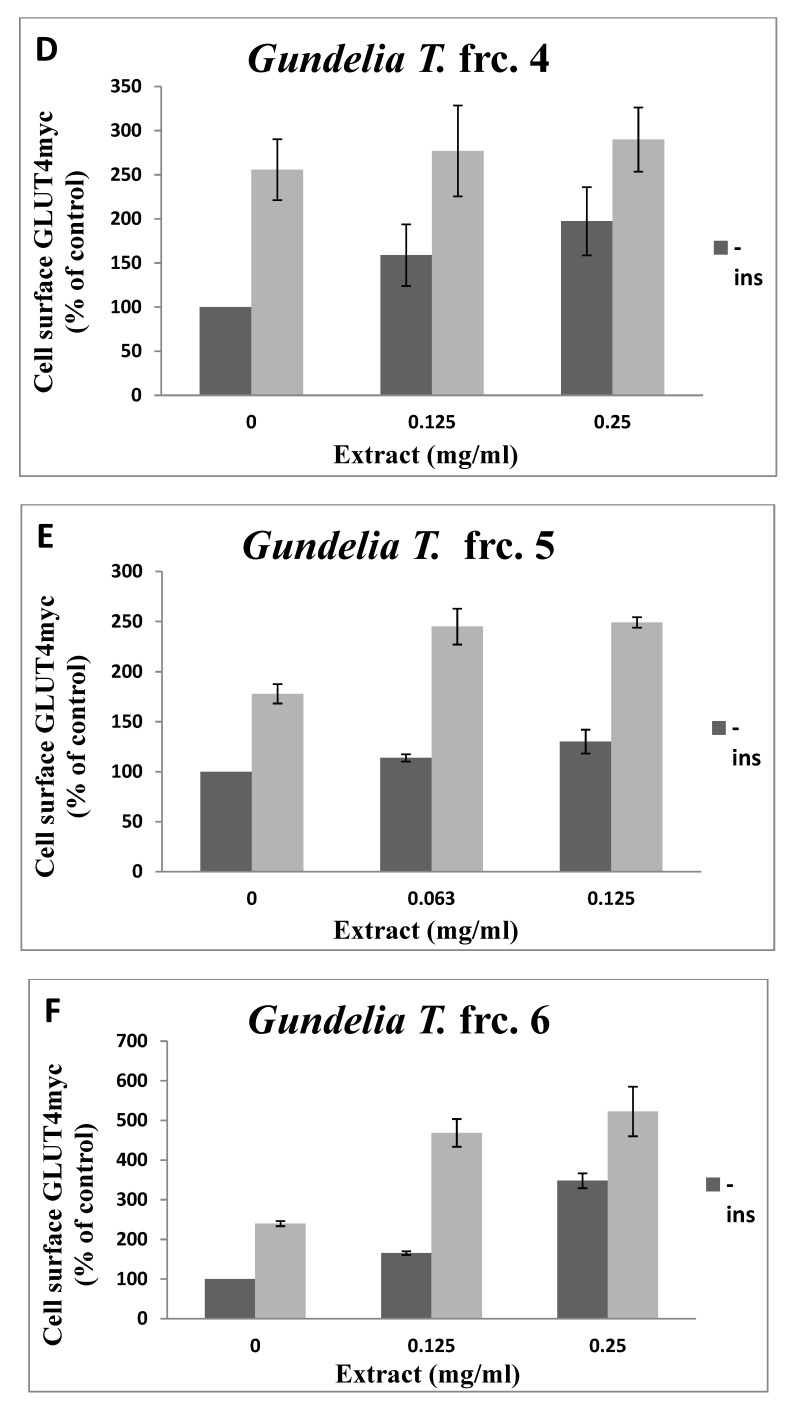

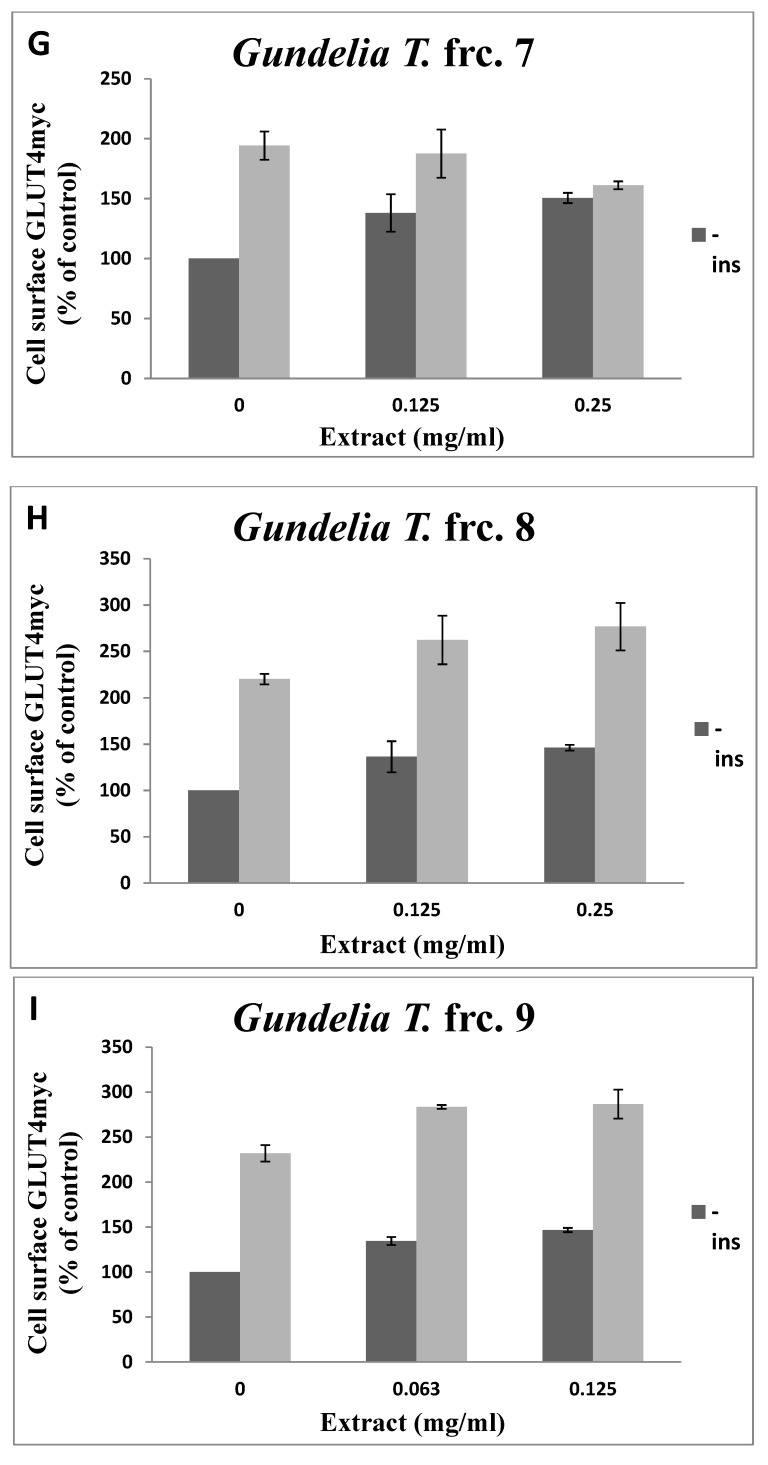

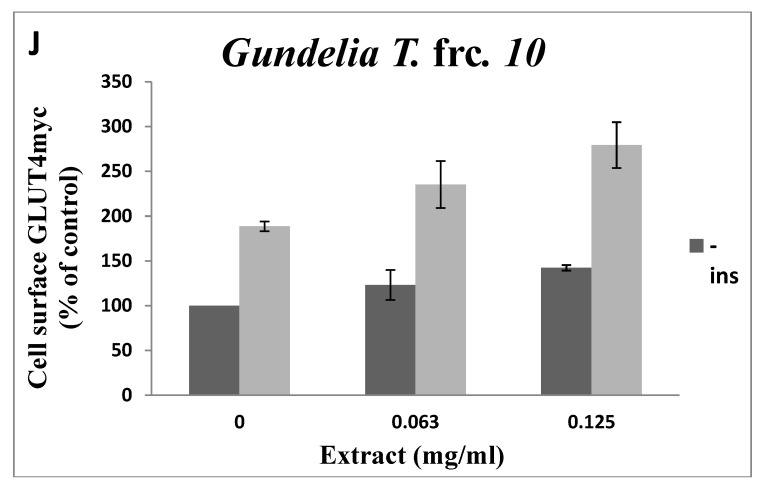
GLUT4 translocation to the plasma membrane. For the evaluation of the GLUT4 L6-GLUT4myc, cells (150,000 cell/well) were exposed to GT fractions (**A**–**J**) for 20 h. Serum depleted cells were treated without (−) or with (+)1 µM insulin for 20 min at 37 °C and surface *myc*-tagged GLUT4 density was quantified using the antibody coupled colorimetric assay. Values given represent means ± SEM (relative to untreated control cells) of three independent experiments carried out in triplicates.

The results indicated that fraction 6 was the most efficient, as it enhanced GLUT4 translocation about 3.5 times in the absence of insulin, and 5.2 times in the presence of insulin when treated with 250 µg/mL. Fractions 1 and 3 had no significant effects on GLUT4 translocation. At 250 µg/mL, fractions 4 and 7 increased GLUT4 translocation 2.0 and 1.5 times, respectively, in the absence of insulin. The other fractions enhanced GLUT4 translocation about 1.3 to 1.5 times in the absence of insulin at 125 µg/mL, and up to 2.9 times in the presence of insulin (Figure 3 and Table 3).

### 3.3. GT Fraction Components Attributed to GLUT4 Translocation

In the first fraction, 18 compounds were detected. Six compounds were identified as glucose disposal enhancers, especially in muscle cells. However, this first fraction did not enhance GLUT4 translocation in L6-GLUT4myc cells. This might have been due to the antagonistic effects of other chemicals. Only three compounds were detected in fraction 2: palmitic acid, β-amyrin, and lupeol. All are known to enhance glucose disposal in muscle except for palmitic acid.

Nine compounds in fraction 3 had anti-diabetic activity (Table 2). However, fraction 3 did not affect GLUT4 translocation to the PM. Most of the nine compounds were present at very low levels (up to 2.5%), except ursolic acid, ursolic aldehyde, and palmitic acid, which accounted for 38.46%, 19.3%, and 8.2%, respectively. Ursolic acid and palmitate are known to augment GLUT4 translocation in muscle cells. Conversely, brucine, a toxic alkaloid, is an inhibitor of the AKT, ERK, and mTOR signaling pathways [15]. It accounted for 11.65% of the fraction and might be one of the major antagonists of GLUT4 translocation enhancers.

Six compounds out of the 14 detected in fraction 4 had anti-diabetic activity (Table 2). Although brucine (believed to inhibit GLUT4 translocation) accounted for half of this fraction, it enhanced GLUT4 translocation 3.5 times at 0.25 mg/mL under basal conditions. This could have been due to the presence of four other compounds known to stimulate GLUT4 translocation in muscle cells, namely, hydroxylamine, myristic acid, palmitic acid, and stearic acid.

Half of the molecules in fraction 5 were considered to be anti-diabetic. A phenolic derivative of benzoic acid, 4-hydroxybenzoic acid, accounted for 76% of the entire fraction. It reportedly reduces plasma glucose levels in streptozotocin-induced diabetic rats, without affecting either serum insulin levels or liver glycogen content [16,17].

Six of the 15 compounds detected in fraction 6, namely 4-hydroxybenzoic acid, isovanillic acid, azelaic acid, quinic acid, palmitic acid, and stearic acid, are known to enhance glucose disposal (Table 2). These active compounds accounted for 48% of the fraction. Furthermore, 33% of all compounds in fraction 7 (six out of 16 compounds) were glucose disposal enhancers. None of the other compounds are known to affect GLUT4 translocation.

In fraction 8, only seven of the 23 detected compounds were associated with anti-diabetic activity. Similarly, only four compounds out of the 16 in fraction 9 had anti-diabetic activity. Remarkably, myo-inositol (which accounted for about 23% of the compounds detected in fraction 8) stimulates GLUT4 translocation to the PM in L6 myotubes and the muscles of mice [18]. Five of the 29 compounds in fraction 10 are known to enhance GLUT4 translocation to the PM (Table 2).

**Table 2:** Phytochemicals of *Gundelia tournefortii* methanol extract fractions verified by gas chromatography–mass spectrometry.

**Fraction 1**						
**Peak**	**Name**	**R_t_**	**% Area**	**Match Factor**	**Association with Diabetes**	**References**
1	Hydroxylamine	15.28	0.60	93.2	Enhances glucose uptake in C_2_C_12_ skeletal muscle cells	[19]
2	Glycerol	20.34	0.10	86.6		
3	Neophytadiene	33.02	0.46	93.3		
4	Myristic acid	33.22	0.08	81.2	Enhances basal glucose uptake in myotubes	[20]
5	3,7,11,15-Tetramethyl-2-hexadecen-1-ol	33.67	0.20	90.8		
6	Methyl palmitate	34.28	1.80	94.6		
7	Palmitic acid	35.81	0.70	95.7	Enhances basal glucose uptake in myotubes	[21,22]
8	9,12-Octadecadienoic acid, methyl ester, (E,E)-	36.34	0.67	93.1		
9	9-Octadecenoic acid (Z)-methyl ester	36.41	0.87	89.4		
10	Methyl stearate	36.68	0.15	88.9		
11	α-Linolenic acid	37.04	0.30	86.3	Enhances insulin secretion from pancreatic beta cells	[23]
12	Stearic acid	37.86	0.43	93.7	Enhances basal glucose uptake in myotubes	[23]
13	Dinonyl phthalate	45.19	0.11	82.2		
14	24-Noroleana-3,12-diene	47.32	0.48	83.7		
15	β-amyrin acetate	54.89	18.74	93.3		
16	Lupeol	55.40	49.25	82.4	Stimulates glucose utilization by skeletal muscles	[24]
17	Cycloartenyl acetate	56.37	8.19	83.3		
18	Lupeol-trifluoroacetate	56.53	16.88	82.8	Stimulates glucose utilization by skeletal muscles	[24]
**Fraction 2**						
**Peak**	**Name**	**R_t_**	**% Area**	**Match Factor**	**Association with Diabetes**	**References**
1	Palmitic acid	35.81	11.2	91.6	Enhances basal glucose uptake in myotubes	[21,22]
2	β-Amyrin	53.60	35.00	90.3	Reduces elevated plasma glucose levels during the oral glucose tolerance test in mice and α-glucosidase inhibitor	[25,26]
3	Lupeol	55.31	53.79	86.1	Stimulates glucose utilization by skeletal muscles	[24]
**Fraction 3**						
**Peak**	**Name**	**R_t_**	**% Area**	**Match Factor**	**Association with Diabetes**	**References**
1	Benzoic acid	19.13	0.19	91.1		
2	Glycerol	20.34	0.11	88.9		
3	4-(Methoxycarbonyl)phenol	25.67	1.88	93.4		
4	Lauric acid	29.39	0.39	82.3	Enhances glucose-stimulated insulin secretion	[27]
5	Azelaic acid	32.45	1.13	88.1	Restores normal levels of plasma glucose, insulin, HbA1c, Hb, liver glycogen, and carbohydrate in diabetic mice	[28]
6	Myristic acid	33.21	0.53	94.6	Enhances basal glucose uptake in myotubes	[20]
7	Pentadecanoic acid	34.61	0.07	86.2		
8	Palmitic acid	35.82	8.22	98.7	Enhances basal glucose uptake in myotubes	[21,22]
9	Heptadecanoic acid	36.88	0.22	86.8		
10	Linoelaidic acid	37.57	1.41	92.8		
11	9-Octadecenoic acid, (E)-	37.62	2.88	96		
12	Stearic acid	37.86	2.56	97.1	Enhances basal glucose uptake in myotubes	[23]
13	Eicosanoic acid	39.61	0.48	88.6		
14	Glyceryl palmitate	40.96	3.54	86		
15	Glycerol monostearate	43.08	1.28	93.5		
16	Lignoceric acid	43.68	0.50	87.1		
17	Stigmasterol	52.84	2.62	94.5	Increases GLUT4 translocation and expression	[29]
18	β-Sitosterol	53.65	2.54	90.9	Improves glycemic control through activation of insulin receptors and GLUT4 in adipose tissue	[30]
19	Ursolic acid	56.32	38.46	91.3	Stimulates glucose uptake in 3T3-L1 adipocytes and α-glucosidase inhibitor	[31]
20	Ursolic aldehyde	56.86	19.33	59.4	Ursolic acid analogs are α-glucosidase inhibitors	[25]
21	Brucine	57.35	11.65	91.7		
**Fraction 4**						
**Peak**	**Name**	**R_t_**	**% Area**	**Match Factor**	**Association with Diabetes**	**References**
1	Hydroxylamine	15.28	0.22	83.9	Enhances glucose uptake in C_2_C_12_ skeletal muscle cells	[19]
2	Glycerol	20.34	0.61	91		
3	Benzeneacetic acid	20.60	2.83	94.7		
4	Suberic acid	30.51	0.46	83.8		
5	Azelaic acid	32.45	5.87	92.1	Restores normal levels of plasma glucose, insulin, HbA1c, Hb, liver glycogen, and carbohydrate in diabetic mice	[28]
6	Myristic acid	33.21	0.64	88.5	Enhances basal glucose uptake in myotubes	[20]
7	Gallic acid	34.99	0.74	81.9		
8	Palmitic acid	35.82	11.79	98.6	Enhances basal glucose uptake in myotubes	[21,22]
9	Linoelaidic acid	37.57	3.04	83.9		
10	Stearic acid	37.86	5.64	95	Enhances basal glucose uptake in myotubes	[23]
11	Glyceryl palmitate	40.96	7.82	90		
12	Glycerol monostearate	43.08	4.77	91.2		
13	Stigmasterol	52.84	2.14	85.1	Increases GLUT4 translocation and expression	[29]
14	Brucine	57.36	53.42	91.7		
**Fraction 5**						
**Peak**	**Name**	**R_t_**	**% Area**	**Match Factor**	**Association with Diabetes**	**References**
1	Lactic Acid	13.71	0.28	86.2		
2	Glycerol	20.34	0.26	92.6		
3	Benzeneacetic acid	20.59	1.10	95.6		
4	4-Hydroxybenzoic acid	28.93	76.20	98.9	Increases glucose consumption in normal and diabetic rats	[16,17]
5	Isovanillic acid	31.89	0.29	83	Stimulates a dose-dependent increase in glucose transport through GLUT4	[32]
6	Azelaic acid	32.45	0.55	89.9	Restores normal levels of plasma glucose, insulin, HbA1c, Hb, liver glycogen, and carbohydrate in diabetic mice	[28]
7	D-Pinitol	33.93	0.39	86.4	Stimulates translocation of GLUT4 in skeletal muscle of C57BL/6 mice and induces translocation of GLUT4 to the plasma membrane	[18,33]
8	Palmitic acid	35.81	3.18	98.6	Enhances basal glucose uptake in myotubes	[21,22]
9	Stearic acid	37.86	1.99	94.7	Enhances basal glucose uptake in myotubes	[23]
10	Glyceryl palmitate	40.96	4.56	96		
11	Glycerol monostearate	43.08	3.45	94.1		
12	Brucine	57.30	7.75	90.2		
**Fraction 6**						
**Peak**	**Name**	**R_t_**	**% Area**	**Match Factor**	**Association with Diabetes**	**References**
1	Lactic Acid	13.69	0.66	92.7		
2	Glycerol	20.34	0.62	91.8		
3	4-Hydroxybenzoic acid	28.89	7.67	98.2	Increases glucose consumption in normal and diabetic rats	[16,17]
4	Isovanillic acid	31.89	8.35	96.8	Stimulates a dose-dependent increase in glucose transport through GLUT4	[32]
5	Azelaic acid	32.45	1.14	92.9	Restores normal levels of plasma glucose, insulin, HbA1c, Hb, liver glycogen, and carbohydrate in diabetic mice	[28]
6	Quinic acid	33.88	4.21	87.7	Enhances glucose-stimulated insulin secretion in both INS-1E cells and mouse islets	[34]
7	Dihydroferulic acid	34.01	2.61	94.2		
8	4-Coumaric acid	34.57	7.73	96.5		
9	Indole-5-carboxylic acid	35.67	16.26	84.9		
10	Palmitic acid	35.81	6.18	98	Enhances basal glucose uptake in myotubes	[21,22]
11	Isoferulic acid	36.42	9.52	94.6		
12	Stearic acid	37.86	5.18	96.3	Enhances basal glucose uptake in myotubes	[23]
13	Glyceryl palmitate	40.95	11.71	96.3		
14	Glycerol monostearate	43.08	9.65	97.1		
15	Questinol	52.85	8.50	82.1		
**Fraction 7**						
**Peak**	**Name**	**R_t_**	**% Area**	**Match Factor**	**Association with Diabetes**	**References**
1	Propanoic acid	16.86	2.74	91.1		
2	Glycerol	20.34	1.40	93.8		
3	Succinic acid	21.20	47.86	98.1		
4	4-Hydroxybenzoic acid	28.89	0.36	87.8	Increases glucose consumption in normal and diabetic rats	[16,17]
5	Azelaic acid	32.45	7.81	94.6	Restores normal levels of plasma glucose, insulin, HbA1c, Hb, liver glycogen, and carbohydrate in diabetic mice	[28]
6	D-Ribonic acid	32.66	1.45	90		
7	Protocatechuic acid	32.94	12.24	95.9	Protects mesangial cells against high glucose damage via inhibition of the p38 MAPK signaling pathway	[35,36,37]
8	Quinic acid	33.88	1.01	83.8	Enhances glucose-stimulated insulin secretion in both INS-1E cells and mouse islets	[34]
9	Syringic acid	34.09	1.49	94.3		
10	Caffeic acid	36.93	12.81	95.8	Reduces insulin resistance and modulates glucose uptake in HepG2 cells	[38]
11	1,2-Hexadecanediol	37.56	5.02	82.7		
12	Stearic acid	37.86	2.03	95.2	Enhances basal glucose uptake in myotubes	[23]
13	Glyceryl palmitate	40.95	1.37	90.3		
14	Chrysophanol	43.05	1.60	85.2	Increases GLUT4 expression in myotubes	[39]
15	Decanedioic acid, bis(2-ethylhexyl) ester	43.27	0.82	81.9		
**Fraction 8**						
**Peak**	**Name**	**R_t_**	**% Area**	**Match Factor**	**Association with Diabetes**	**References**
1	Hydroxylamine	15.28	0.28	90.8	Enhances glucose uptake in C_2_C_12_ skeletal muscle cells	[19]
2	Hydracrylic acid	16.33	1.65	94.7		
3	Glycerol	20.34	0.46	93.7		
4	Succinic acid	21.22	42.22	98.3		
5	Uracil	21.92	14.05	97.1		
6	5-Methylpyrimidine-2,4-diol	23.59	5.37	91.8		
7	4,5-Dihydro-4-hydroxy-5-(hydroxymethyl)-2(3H)-furanone	26.22	7.73	86.3		
8	Pyroglutamic acid	26.52	2.87	83.2	Reduces oral glucose tolerance and serum insulin levels in rats	[40]
9	3,4,5-Trihydroxytetrahydro-2H-pyran-2-one	29.22	4.04	87.1		
10	3,4-Dihydroxy-5-(hydroxymethyl)dihydrofuran-2(3H)-one	29.31	1.34	93.6		
11	D-(+)-Ribono-1,4-lactone	30.39	0.77	89		
12	Xylonic acid	32.65	0.70	90.9		
13	Protocatechuic acid	32.94	0.76	91.7	Protects mesangial cells against high glucose damage via inhibition of the p38 MAPK signaling pathway	[35,36,37]
14	Quinic acid	33.89	1.70	84.9	Enhances glucose-stimulated insulin secretion in both INS-1E cells and mouse islets	[34]
15	Gulonic acid gamma-lactone	34.33	1.34	83.5		
16	D-Gluconic acid	35.76	1.84	92.9		
17	Caffeic acid	36.93	0.66	92.9	Reduces insulin resistance and modulates glucose uptake in HepG2 cells	[38]
18	Stearic acid	37.86	1.67	96.7	Enhances basal glucose uptake in myotubes	[23]
19	Glyceryl palmitate	40.95	0.83	86.4		
20	Glycerol monostearate	43.08	0.64	84.1		
21	Decanedioic acid, bis(2-ethylhexyl) ester	43.28	0.97	89.9		
22	Genistein	50.21	0.26	83.4	Improves insulin secretion from pancreatic beta cells	[41]
23	Brucine	57.30	7.84	91.6		
**Fraction 9**						
**Peak**	**Name**	**R_t_**	**% Area**	**Match Factor**	**Association with Diabetes**	**References**
1	Hydracrylic acid	16.34	0.44	93		
2	Urea	19.16	2.42	95.1		
3	Glycerol	20.37	35.50	97.8		
4	Butanedioic acid	21.20	1.98	97.7		
5	Meso-erythritol	26.60	1.97	97.8		
6	2-Isopropylmalic acid	27.99	0.67	92.4		
7	2-Deoxy-D-ribitol	28.73	0.73	93		
8	Quinic acid	33.89	7.75	89.4	Enhances glucose-stimulated insulin secretion in both INS-1E cells and mouse islets	[34]
9	D-(-)-Fructose	34.11	8.16	95.9		
10	L-(-)-Sorbose	34.25	4.30	96		
11	D-Sorbitol	34.88	4.79	98		
12	Myo-Inositol	35.18	22.82	96.3	Stimulates translocation of GLUT4 in skeletal muscle of C57BL/6 mice and induces translocation of GLUT4 to the plasma membrane	[18,33]
13	D-Gluconic acid	35.76	0.75	91.5		
14	Caffeic acid	36.93	1.71	95.1	Reduces insulin resistance and modulates glucose uptake in HepG2 cells	[38]
15	D-(+)-Trehalose	43.33	3.94	95.1		
16	Chlorogenic acid	50.61	2.06	80.5	Reduces insulin resistance and modulates glucose uptake in HepG2 cells	[38]
**Fraction 10**						
**Peak**	**Name**	**R_t_**	**% Area**	**Match Factor**	**Association with Diabetes**	**References**
1	L-Proline	16.99	4.91	91.8		
2	L-Valine	18.50	1.11	95.5		
3	Urea	19.08	0.69	96.4		
4	L-Leucine	20.14	0.08	90.1		
5	Glycerol	20.34	2.69	97.3		
6	Butanedioic acid	21.19	0.43	96.1		
7	Glyceric acid	21.88	0.99	95.8		
8	Serine	22.66	0.32	95.3		
9	L-Threonine	23.37	0.71	90.7		
10	3-Aminoisobutyric acid	25.01	0.26	91.9		
11	Pyroglutamic acid	26.52	3.94	96.7	Reduces oral glucose tolerance and serum insulin levels in rats	[40]
12	4-Aminobutanoic acid	26.75	3.15	94.8		
13	Threonic acid	27.44	0.25	92.6		
14	L-Threonic acid	27.86	0.31	93.3		
15	Phenylalanine	28.98	0.67	96.1		
16	Asparagine	30.11	0.18	85.2		
17	D-(+)-Arabitol	31.00	0.35	88.8		
18	Xylitol	31.00	0.35	87.4		
19	Quinic acid	33.90	58.03	89.9	Enhances glucose-stimulated insulin secretion in both INS-1E cells and mouse islets	[34]
20	D-(-)-Fructose	34.11	2.16	91.8		
21	L-(-)-Sorbose	34.25	0.66	93.8		
22	D-(+)-Talose	34.45	0.37	86.1		
23	L-Tyrosine	34.72	0.32	85		
24	D-Sorbitol	34.87	0.92	96.9		
25	Myo-inositol	35.18	12.01	96	Stimulates translocation of GLUT4 in skeletal muscle of C57BL/6 mice and induces translocation of GLUT4 to the plasma membrane	[18,33]
26	D-Gluconic acid	35.81	0.70	84.9		
27	Caffeic acid	36.93	0.67	94	Reduces insulin resistance and modulates glucose uptake in HepG2 cells	[38]
28	D-(+)-Trehalose	43.33	4.34	96.2		
29	Chlorogenic acid	50.61	1.56	80.8	Reduces insulin resistance and modulates glucose uptake in HepG2 cells	[38]

HbA1c, glycated hemoglobin; Hb, hemoglobin; GLUT4, glucose transporter type 4.

**Table 3:** Summary of the cytotoxicity and anti-diabetic activity of GT fractions.

Fraction Number	Cytotoxicity (µg/mL), Safe Up to:	GLUT4 Translocation (% Relative to Controls) at 125 µg/mL	
		− Insulin Relative to Control without Insulin	+ Insulin Relative to Control with Insulin
1	500	1.14	0.94
2	500	1.18	1.40
3	500	1.10	1.00
4	250	1.59	1.08
5	125	1.30	1.40
6	250	1.65	1.95
7	250	1.38	0.96
8	500	1.36	1.19
9	500	1.46	1.24
10	500	1.42	1.48

GT, *Gundelia tournefortii*; GLUT4, glucose transporter type 4.

## 4. Discussion

Phytochemicals and herbal extracts have become major factors of drug development programs, particularly because of minimal costs and fewer adverse effects [42]. Indeed, anti-diabetic herbal drugs are usually effective and lead to fewer side effects [43]. Recently, we reported that GT methanol extract could efficiently promote GLUT4 translocation to the PM of L6 muscle cells [9].

In the current study, GT methanol extract was sub-fractioned into 10 samples via flash chromatography. We detected 25 distinct anti-diabetic molecules, among which the major molecules were: lupeol (fractions 1 and 2); lupeol-trifluoroacetate (fraction 1); palmitic acid (fractions 2 and 4); β-amyrin (fraction 2); ursolic acid and ursolic aldehyde (fraction 3); 4-hydroxybenzoic acid (fraction 5); 3,4-dihydroxybenzoic acid and caffeic acid (fraction 7); myo-inositol (fractions 9 and 10); and quinic acid (fraction 10). All fractions (except 1 and 3) were able to efficiently stimulate GLUT4 translocation to the PM of L6-GLUT4myc cells. The translocation of GLUT4 to the PM is the main process that accelerates glucose uptake into cells, in response to insulin or other stimuli [7].

### 4.1. Lupeol and Lupeol-Trifluoroacetate

Lupeol and lupeol derivatives increased GLUT4 translocation to the PM in L6 muscle cells, by up to 2.0 times [24]. Lupeol stimulation of glucose uptake is associated with the activation of the IRS-1/PI3K/AKT-dependent signaling pathway in L6 cells, which leads to enhanced translocation of GLUT4 [24]. Although lupeol and lupeol-trifluoroacetate were both present in fraction 1, no significant effects on GLUT4 translocation were observed. This might have been due to the antagonistic effects of other chemicals. For instance, methyl palmitate opens K channels [44], which in turn reduces GLUT4 translocation to the PM [45]. Lupeol also accounted for 11.2% of fraction 2. This fraction contained only three compounds, all of which are known to promote GLUT4 translocation to the PM (Table 2). Interestingly, lupeol can reportedly bind directly to GLUT4 [46], and this action might enhance its activation of the IRS-1/PI3K/AKT-dependent signaling pathway [24].

### 4.2. Palmitic Acid

Palmitic acid was present in fractions 1 to 6, but was observed at high concentrations in fractions 2 and 4 alone (about 11%). Glucose uptake is enhanced in skeletal muscle treated with 300 µM palmitic acid for up to 60 min [22]. Similarly, short-term treatment of adipocytes with fatty acids increases basal glucose uptake [47,48]. However, long-term exposure (16–24 h) of myocytes to palmitate induces insulin resistance [21,49,50].

In the current study, L6 cells were exposed to GT fractions for 23 h and palmitic acid accounted for 0.7%, 11.2%, 8.2%, 11.8%, 3.2%, and 6.2% in fractions 1 to 6, respectively. The presence of relatively high levels of palmitic acid in fractions 2, 3, and 4 could explain the relatively lower translocation levels observed in those fractions. In fraction 2, only three compounds were detected: β-amyrin, lupeol, and palmitic acid. The first two compounds are known to enhance glucose uptake and GLUT4 translocation [26,51,52]. However, GLUT4 translocation in L6 cells treated with 250 µg/mL of fraction 2 was increased by only 1.3 times in the presence of insulin and 1.22 times in the absence of insulin (relative to the insulin control). This could be attributed to the negative effects of palmitic acid.

Fraction 3 did not affect GLUT4 translocation, while fraction 4 increased GLUT4 translocation by 1.6 times at 125 µg/mL and 2.0 times at 250 µg/mL, in the absence of insulin. No significant effects were observed in the presence of insulin (Figure 3. These results could be attributed to the high levels of brucine (50.5%). Brucine is an inhibitor of the AKT, ERK, and mTOR signaling pathways [15] and might be a major antagonist of GLUT4 translocation enhancers.

Brucine accounted for 11.6%, 53.4%, 7.7%, and 7.8% of fractions 3, 4, 5, and 8. The present findings might explain why the highest levels of GLUT4 translocation were observed in L6 cells treated with fraction 6 (3.5 times basal levels in the absence of insulin, and 5.2 times basal levels in the presence of insulin, when treated with 250 µg/mL; Figure 3). These results could also be attributed to the high levels of GLUT4 activators in fraction 6, as six out of the 15 compounds detected in that fraction are known to enhance glucose disposal. Those six compounds were 4-hydroxybenzoic acid, isovanillic acid, azelaic acid, quinic acid, palmitic acid, and stearic acid (Table 2). These active compounds accounted for 32.8% of fraction 6.

### 4.3. β-Amyrin

Fraction 2 was the only fraction in which β-amyrin was detected (35%), and β-amyrin acetate accounted for 18.7% of fraction 1. A previous study reported that β-amyrin can reduce the elevated plasma glucose levels in streptozotocin-induced diabetic mice during an oral glucose tolerance test [26]. In an independent study, β-amyrin palmitate led to similar results in diabetic rats [52].

Interestingly, membrane GLUT4 levels and glucose uptake in 3T3-L1 adipocytes treated with β-amyrin were significantly higher than in control cells [51]. Therefore, the increased GLUT4 translocation observed in cells treated with fraction 2 could be attributed, at least in part, to β-amyrin. To the best of our knowledge, β-amyrin acetate does not affect glucose disposal nor GLUT4 translocation. This could partially explain the neutral effects of fraction 1 on GLUT4 translocation.

### 4.4. Ursolic Acid and Ursolic Aldehyde

Ursolic acid and ursolic aldehyde were detected in fraction 3 alone, accounting for 38.5% and 19.3%, respectively. Some studies have shown that ursolic acid inhibits protein tyrosine phosphatase 1B (PTP1B), a negative regulator of insulin signaling, and improves insulin sensitivity [53,54]. Ursolic acid combined with rosiglitazone improves insulin sensitivity by increasing skeletal muscle insulin-stimulated IRS-1 tyrosine phosphorylation in diabetic mice fed a high-fat diet [55].

Remarkably, ursolic acid stimulates GLUT4 translocation and glucose uptake in 3T3-L1 adipocytes through the PI3K pathway [31]. Ursolic acid derivatives reportedly inhibit α-glucosidase activity [25]. Other ursolic acid derivatives inhibit intestinal glucose uptake in Caco-2 cells and stimulate insulin secretion in diabetic rats [56]. Even though ursolic acid is known to enhance GLUT4 translocation, fraction 3 showed no significant effects on GLUT4. This could be attributed to the presence of brucine, an inhibitor of the AKT, ERK, and mTOR signaling pathways [15], which likely prevents GLUT4 translocation to the PM.

### 4.5. 4-Hydroxybenzoic Acid and 3,4-Dihydroxybenzoic Acid

In fraction 5, 4-hydroxybenzoic acid accounted for 76.2%; in fraction 6, 7.7%; and in fraction 7, 0.36%. Oral administration of 4-hydroxybenzoic acid lowers blood glucose levels in streptozotocin-induced diabetic rats and normal rats [16,17]. However, its activity could be attributed mainly to the stimulation of insulin secretion [16,17], rather than the direct activation of glucose transporters. This could explain the low levels of GLUT4 translocation in cells exposed to fraction 5, which mainly comprised 4-hydroxybenzoic acid.

The high efficiency of fraction 6 was not related to the presence of 4-hydroxybenzoic acid. One derivative of this compound, 3,4-dihydroxybenzoic acid, also known as protocatechuic acid, accounted for 12.2% of fraction 7. Interestingly, protocatechuic acid is known to reverse hyperglycemia in diabetic rats [57]. Notably, it mimics insulin as it activates the INSR/PI3K/AKT and AMPK pathways, both in vitro and in vivo. It also triggers glucose uptake through GLUT4 translocation [36]. Therefore, we attributed the enhancement of GLUT4 translocation in fraction 7 in part to protocatechuic acid.

### 4.6. Caffeic Acid

Caffeic acid accounted for 12.8% of fraction 7, and 1% of fractions 8, 9, and 10 each. It is associated with the phosphorylation of AKT [58], an upstream activator of GLUT4 translocation. Caffeic acid also attenuates insulin resistance and modulates glucose uptake in HepG2 cells [38]. Those findings are consistent with the present results, as fraction 5 induced a significant increase in GLUT4 translocation. The activity of fractions 8 to 10 cannot be attributed to caffeic acid, owing to its low levels in those fractions.

### 4.7. Myo-Inositol and Quinic Acid

Myo-inositol accounted for 22.8% of fraction 9 and 12.01% of fraction 10. Inositol derivatives stimulate the uptake of glucose, accompanied by the translocation of GLUT4 to the PM in L6 myotubes. Myo-inositol increases GLUT4 translocation in the skeletal muscles of mice and lowers postprandial blood glucose levels [18]. It also activates the AMPK pathway and increases GLUT4 expression [59], leading to GLUT4 translocation and cumulative glucose uptake. These findings are consistent with our observations in fractions 9 and 10, both of which activated GLUT4 translocation. Quinic acid was present in fractions 6 to 10. However, its anti-diabetic activity is associated with the promotion of insulin secretion from pancreatic beta cells [34].

In summary, 25 out of the 98 distinct compounds detected in the 10 GT fractions under investigation showed anti-diabetic activity. Most of those compounds enhanced glucose disposal and GLUT4 translocation to the PM. These active compounds accounted for about 25% of the total number of compounds detected. In addition to the main compounds discussed above, 14 other chemicals present at low levels also showed anti-diabetic properties (Table 2).

## 5. Conclusions

The GT methanol extract sub-fractions (except fractions 1 and 3) significantly stimulated GLUT4 translocation to the PM of L6 myocytes. Among the compounds detected, 25% are reportedly anti-diabetic agents, while 20% are known to enhance either GLUT4 transport or translocation to the PM. The activity of these fractions should be examined in diabetic animal models and human subjects before they can be prescribed as anti-diabetic therapies.

## Figures and Tables

**Table 1:** Gradient conditions.

Hexane	EtoAc	EtOH	Time (min)
From 0%	To 100%		30.2
	100%		15
	From 0%	To 100%	15
		100%	15

EtoAc, ethyl acetate; EtOH, ethanol.

## Data Availability

All data supporting the results are presented in the paper and can be made available from the corresponding author upon reasonable request.

## Supplementary Materials

Supplementary material Figure S1: GC-MS chromatograms of the *Gundelia tournefortii* fractions (frc. 1- frc. 10). Major peaks are labeled with the compounds identified. Zoom; region of the elution of some compounds.
