# Supplementary material for: Gundelia tournefortii: Fractionation, Chemical Composition and GLUT4 Translocation Enhancement in Muscle Cell Line
Source: Molecules. 2021 Jun 22;26(13):3785. doi: 10.3390/molecules26133785 (PMC8270329; doi:10.3390/molecules26133785)
Supplement: Supplementary file 1 [file molecules-26-03785-s001.zip › molecules-1251197-supplementary.pdf]

Supplementary material Fig. 1: GC-MS chromatograms of the *Gundelia tournefortii* fractions (frc. 1- frc. 10). Major peaks are labeled with the compounds identified. Zoom; region of the elution of some compounds.

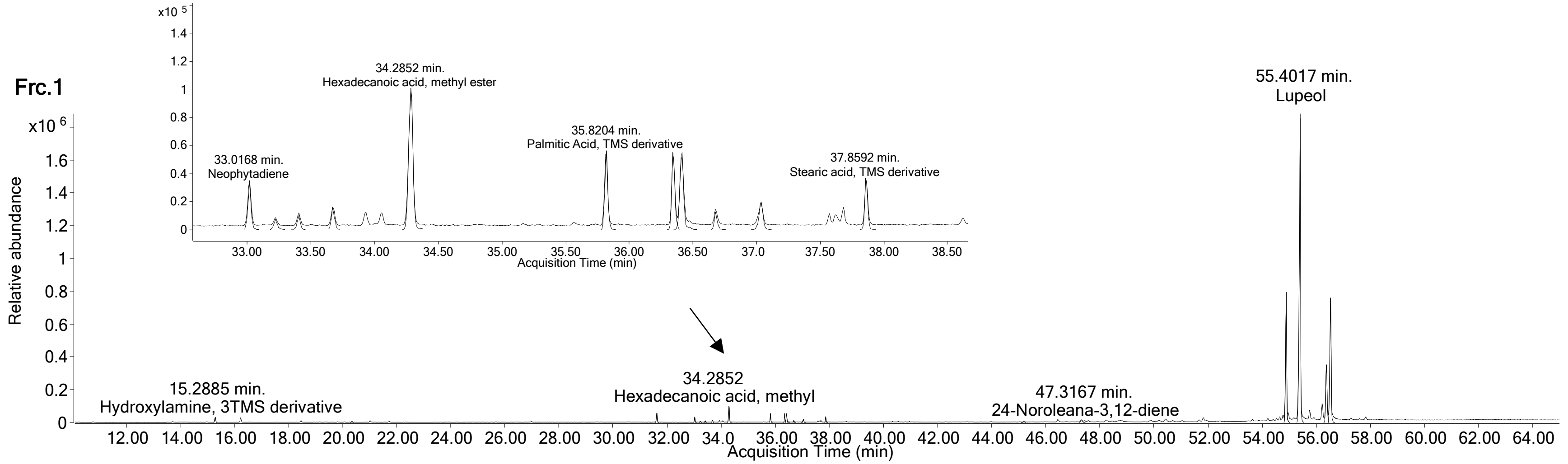

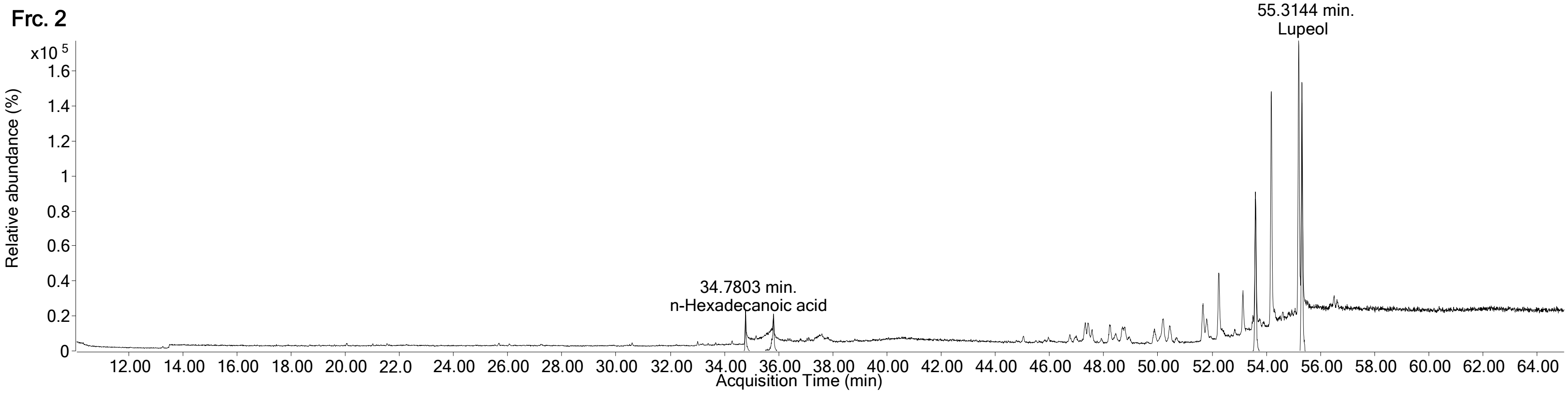

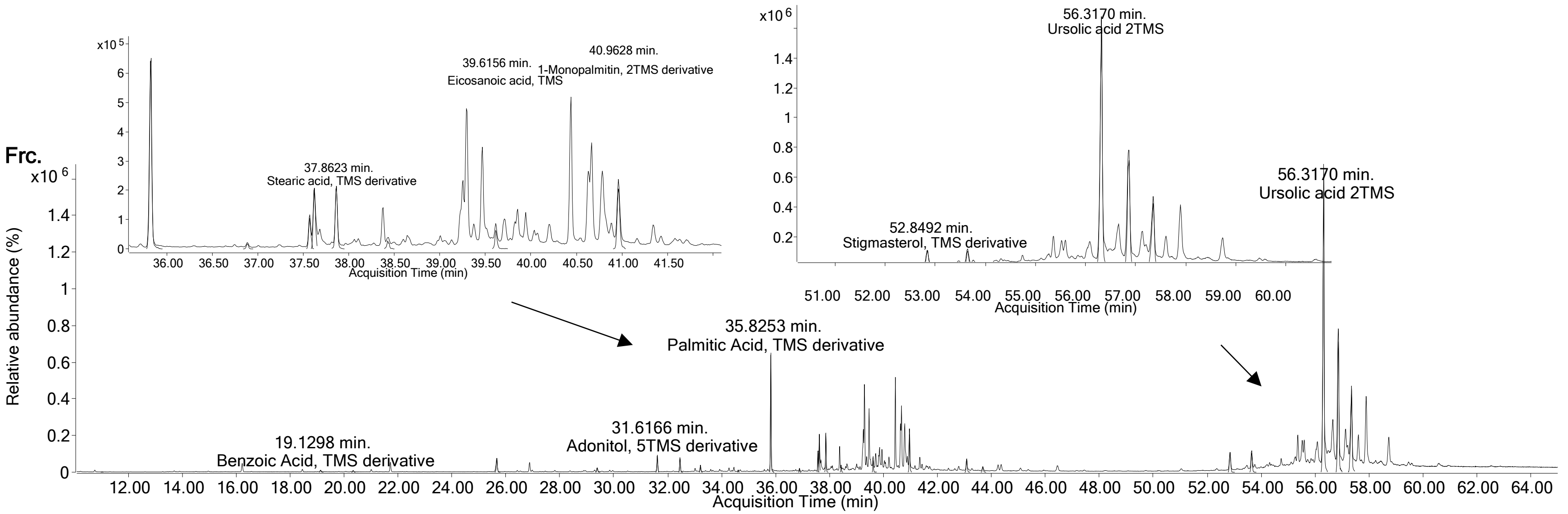

Frc.
x10 6
Relative abundance (%)
Acquisition Time (min)
19.1298 min.
Benzoic Acid, TMS derivative
31.6166 min.
Adonitol, 5TMS derivative
35.8253 min.
Palmitic Acid, TMS derivative
56.3170 min.
Ursolic acid 2TMS
37.8623 min.
Stearic acid, TMS derivative
39.6156 min.
Eicosanoic acid, TMS
40.9628 min.
1-Monopalmitin, 2TMS derivative
52.8492 min.
Stigmasterol, TMS derivative

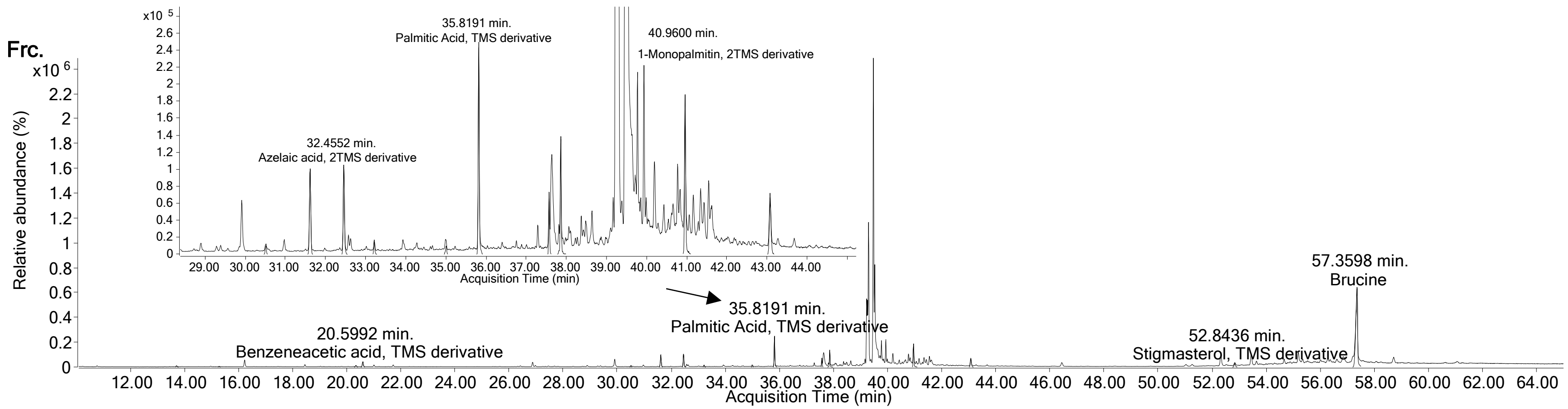

Frc.
x10 6
Relative abundance (%)
Acquisition Time (min)
20.5992 min.
Benzeneacetic acid, TMS derivative
35.8191 min.
Palmitic Acid, TMS derivative
52.8436 min.
Stigmasterol, TMS derivative
57.3598 min.
Brucine
32.4552 min.
Azelaic acid, 2TMS derivative
40.9600 min.
1-Monopalmitin, 2TMS derivative

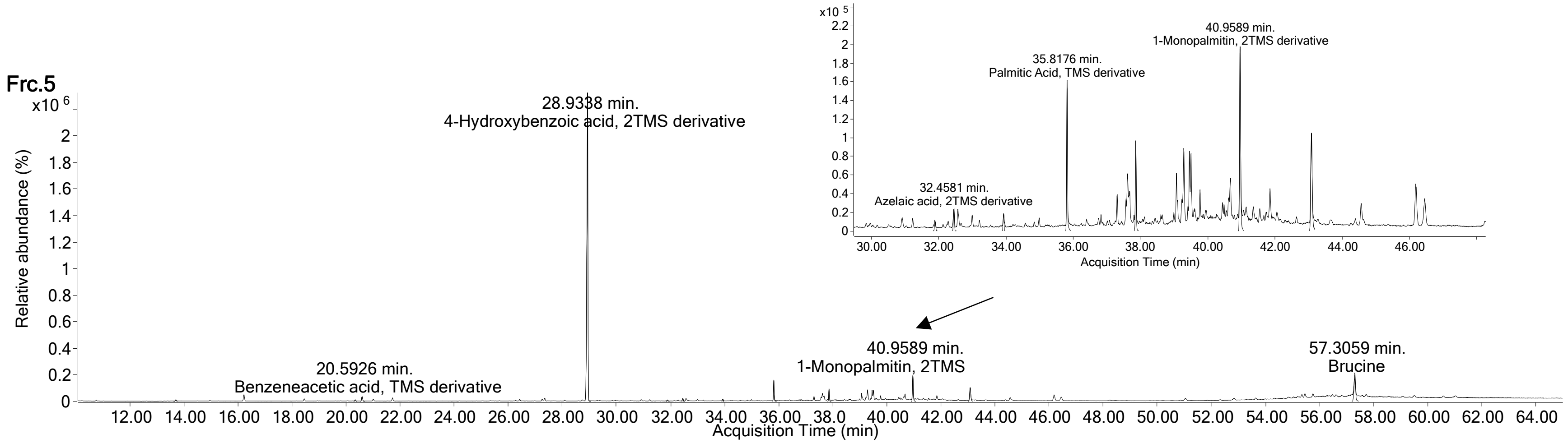
Frc.5
x10 6
Relative abundance (%)
Acquisition Time (min)
28.9338 min.
4-Hydroxybenzoic acid, 2TMS derivative
20.5926 min.
Benzeneacetic acid, TMS derivative
40.9589 min.
1-Monopalmitin, 2TMS
57.3059 min.
Brucine
x10 5
32.4581 min.
Azelaic acid, 2TMS derivative
35.8176 min.
Palmitic Acid, TMS derivative
40.9589 min.
1-Monopalmitin, 2TMS derivative

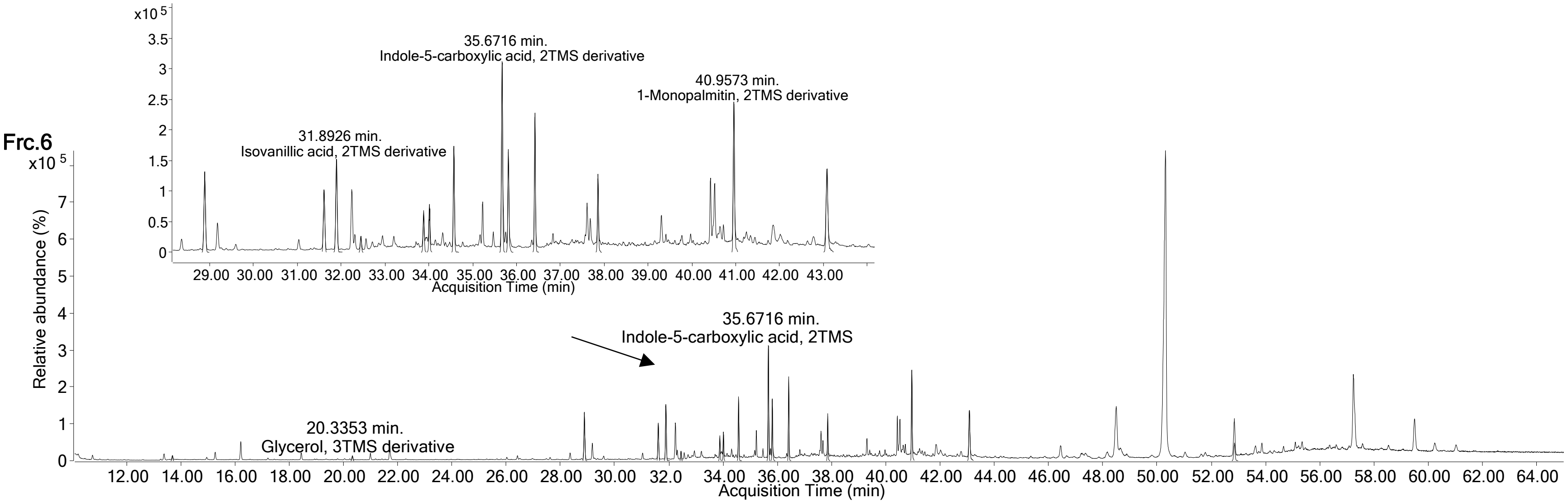
Frc.6
x10 5
Relative abundance (%)
Acquisition Time (min)
20.3353 min.
Glycerol, 3TMS derivative
35.6716 min.
Indole-5-carboxylic acid, 2TMS
x10 5
31.8926 min.
Isovanillic acid, 2TMS derivative
35.6716 min.
Indole-5-carboxylic acid, 2TMS derivative
40.9573 min.
1-Monopalmitin, 2TMS derivative

Frc.7

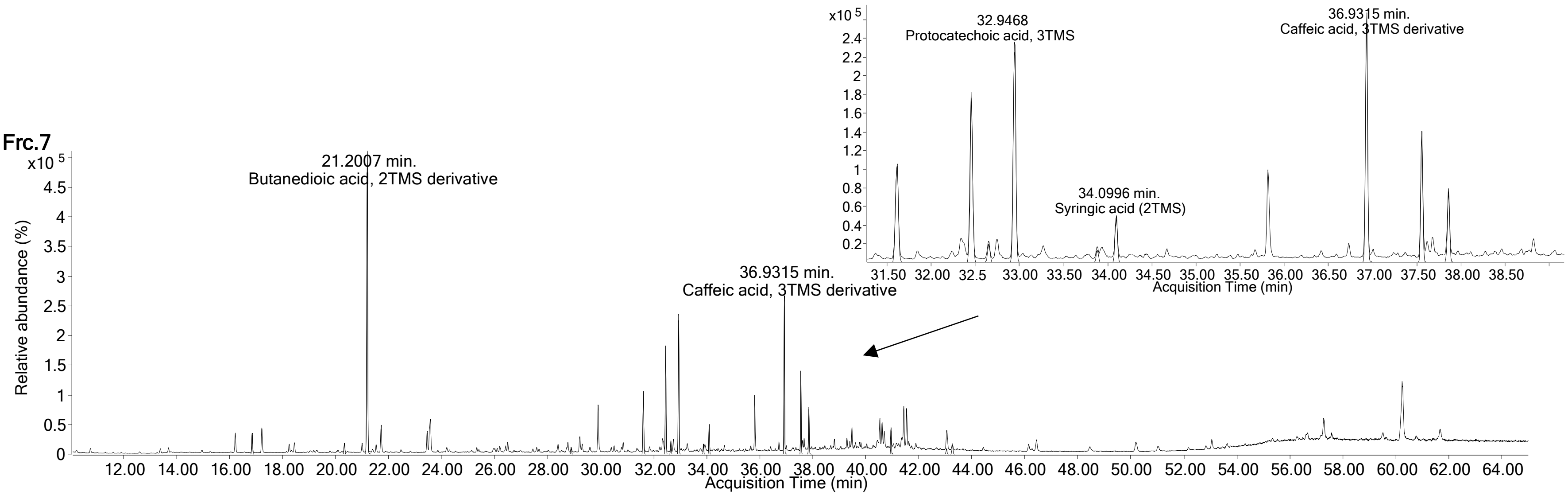
x10 5
Relative abundance (%)
Acquisition Time (min)
21.2007 min.
Butanedioic acid, 2TMS derivative
36.9315 min.
Caffeic acid, 3TMS derivative
32.9468
Protocatechoic acid, 3TMS
36.9315 min.
Caffeic acid, 3TMS derivative
34.0996 min.
Syringic acid (2TMS)
Acquisition Time (min)

Frc.8

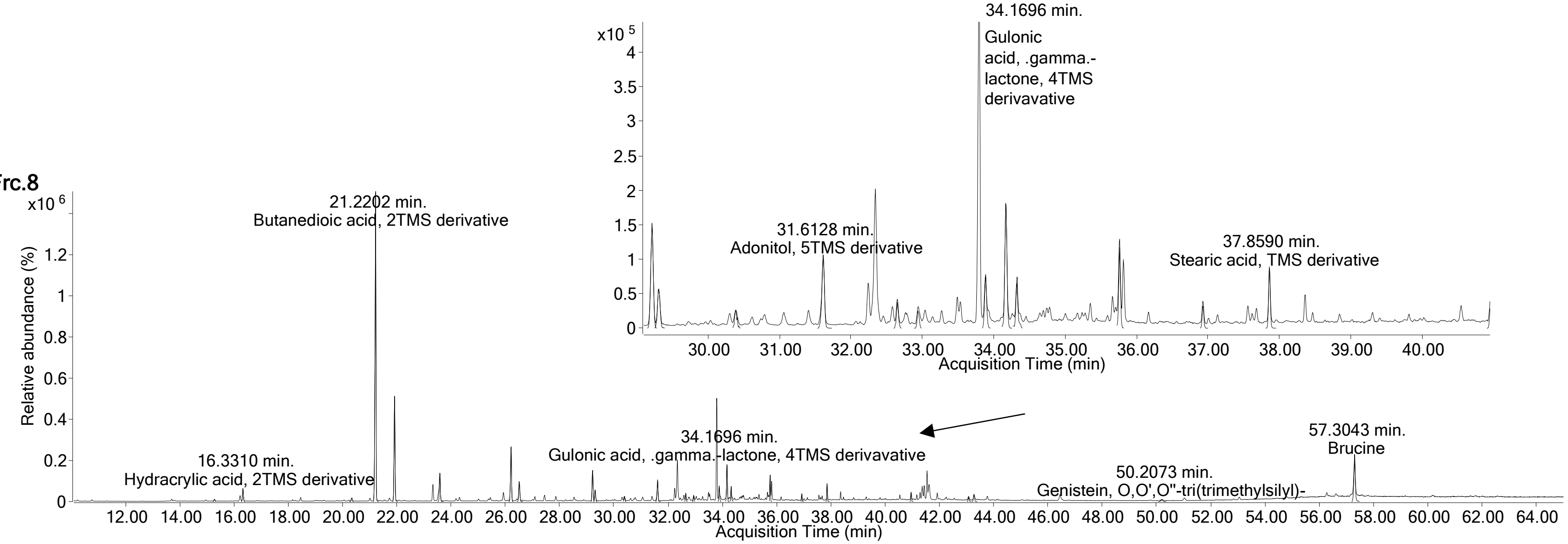
x10 6
Relative abundance (%)
Acquisition Time (min)
21.2202 min.
Butanedioic acid, 2TMS derivative
16.3310 min.
Hydracrylic acid, 2TMS derivative
34.1696 min.
Gulonic acid, .gamma.-lactone, 4TMS derivavative
50.2073 min.
Genistein, O,O',O''-tri(trimethylsilyl)-
57.3043 min.
Brucine
x10 5
34.1696 min.
Gulonic
acid, .gamma.-
lactone, 4TMS
derivavative
31.6128 min.
Adonitol, 5TMS derivative
37.8590 min.
Stearic acid, TMS derivative
Acquisition Time (min)

Frc.9

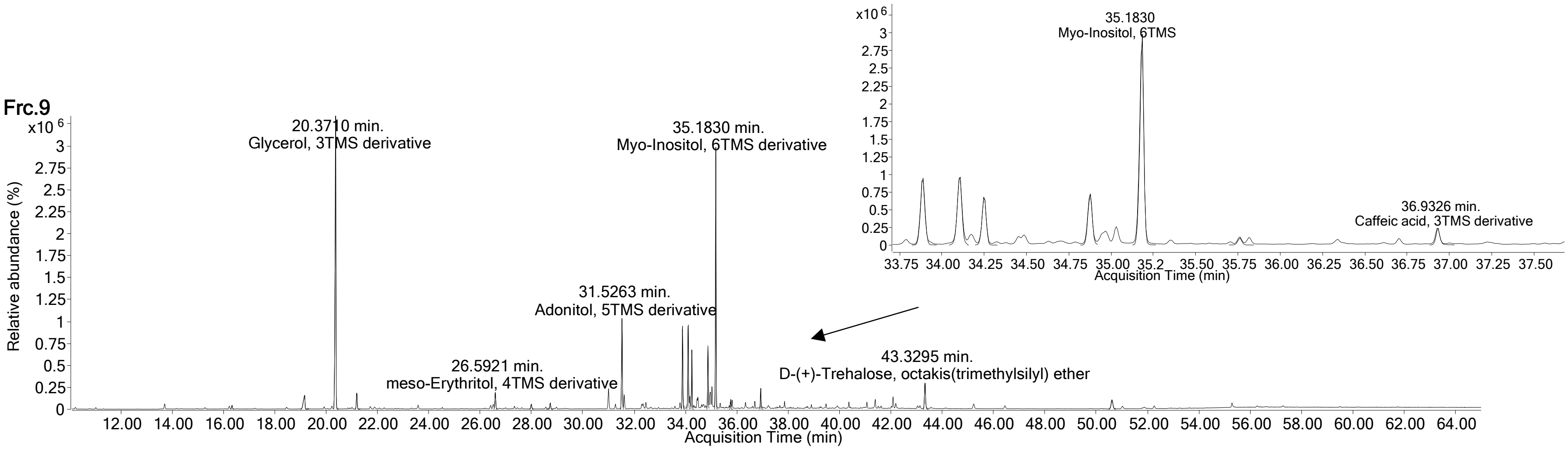
20.3710 min.
Glycerol, 3TMS derivative
35.1830 min.
Myo-Inositol, 6TMS derivative
31.5263 min.
Adonitol, 5TMS derivative
26.5921 min.
meso-Erythritol, 4TMS derivative
43.3295 min.
D-(+)-Trehalose, octakis(trimethylsilyl) ether
35.1830
Myo-Inositol, 6TMS
36.9326 min.
Caffeic acid, 3TMS derivative
Relative abundance (%)
Acquisition Time (min)

Frc.10

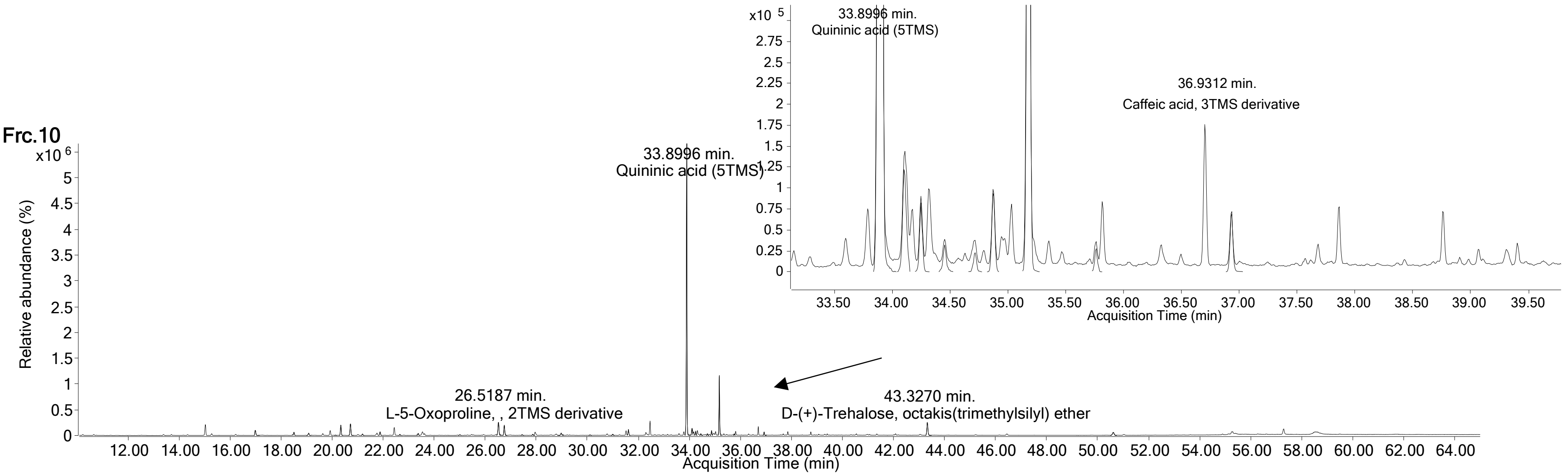
33.8996 min.
Quininic acid (5TMS)
26.5187 min.
L-5-Oxoproline, , 2TMS derivative
43.3270 min.
D-(+)-Trehalose, octakis(trimethylsilyl) ether
36.9312 min.
Caffeic acid, 3TMS derivative
Relative abundance (%)
Acquisition Time (min)
